# HISTORICAL CHANGE IN THE HEALTH AND WELL-BEING OF GHANIAN MIDDLE-AGED AND OLDER ADULTS

**DOI:** 10.1093/geroni/igae098.0443

**Published:** 2024-12-31

**Authors:** Nutifafa Dey, Frank Infurna

**Affiliations:** Arizona State University, Tempe, Arizona, United States; Arizona State University, Tempe, Arizona, United States

## Abstract

The last 70 years in Ghana has seen political and socioeconomic transformations that have impacted adult life through the types of policies implemented and social programs made accessible. It has not yet been studied whether these transformations have led to better health and well-being for middle-aged and older adults in Ghana. Up to this point, studies examining historical change or cohort differences are largely from high-income nations and report trends of historical improvements in health and well-being for middle-aged and older adults in European nations, compared to middle-aged and older adults in the U.S. where historical declines are largely observed. Our goal is to use data from Ghana’s WHO Study on Global Ageing and Adult Health (SAGE) to fill this gap. Multilevel models were applied to three waves of data collected between 2002-2015 and the outcomes were self-rated health and depressive symptoms. Results revealed that later-born cohorts are reporting poorer self-rated health, which is indicative of historical declines. This trend did not differ across women and educational attainment. No significant cohort effects or modifying effects of gender and education were found for depressive symptoms. These findings shed light on the unique historical trajectory of self-rated health and depressive symptoms in middle-aged and older adults in Ghana. Our findings show similarities and differences to previous findings from the U.S. and Europe. Future research is required that tests potential mechanisms, such as changing dynamics in employment, financial demands, and disruptions in traditional family and social life due to modernization and urbanization.

